# Anterior cervical discectomy and fusion with stand-alone anchored cages versus posterior laminectomy and fusion for four-level cervical spondylotic myelopathy: a retrospective study with 2-year follow-up

**DOI:** 10.1186/s12891-018-2136-1

**Published:** 2018-07-12

**Authors:** Bing Wang, Guohua Lü, Lei Kuang

**Affiliations:** Department of Spine Surgery, The Second Xiangya Hospital of Central South University, Changsha City, China

**Keywords:** Cervical spondylopathy, Stand-alone anchored cage, Anterior cervical discectomy and fusion, Retrospective study

## Abstract

**Background:**

The optimal treatment for multi-level cervical spondylotic myelopathy (CSM) remains controversial. Posterior approach is most commonly used, but complicated with insufficient decompression and postoperative axial neck pain. The anterior approach is effective in neural decompression with less surgical trauma. However, the profile of the plate or the possible construct failure may cause dysphagia after surgery. Recently, anterior cervical discectomy and fusion (ACDF) with self-anchored cage is reported to have a superior result over ACDF with anterior plates and screws in three-level CSM. The purpose of the study is to compare the clinical and radiological outcomes of ACDF using stand-alone anchored cages to that of laminectomy with fusion (LF) for treating four-level CSM.

**Methods:**

Twenty-six patients underwent four-level ACDF (Group A) and 32 patients with four-level LF (Group B) were retrospectively reviewed and followed-up for 24 months. Clinical efficacy was evaluated by comparing pre- and post-operative Japanese Orthopedic Association (JOA) and Neck Disability Index (NDI) scores. Operative time, blood loss, fusion, lordosis change and complications were evaluated.

**Results:**

There was significantly less blood loss in Group A (163.4 ± 72.1 ml) than Group B (241.0 ± 112.3 ml) (*P* < 0.05). Both groups demonstrated significant improvements in JOA and NDI scores after surgery with similar operative time. Improvements in cervical lordosis and fused segment lordosis were more pronounced in Group A (11.3 ± 5.9°, 9.7 ± 5.3°) than Group B (5.8 ± 4.6°, 5.5 ± 4.5°) (*P* < 0.05). Loss of lordosis in the cervical spine and fused segment was more prominent in Group A (11.7 ± 2.2°, 6.7 ± 3.2°) than Group B (7.5 ± 3.8°, 3.7 ± 3.4°) (*P* < 0.05) at the final follow-up. Complication rate in Group A and Group B was 57.69 and 18.75%, respectively.

**Conclusions:**

ACDF using a stand-alone anchored cage showed similar clinical results to LF for the treatment of four-level CSM, with better lordosis correction and less blood loss. However, ACDF was associated with more loss of lordosis after surgery and more non-unions.

## Background

Cervical spondylotic myelopathy (CSM) is a clinically symptomatic degenerative condition resulting from the compression of the spinal cord. The optimal treatment for multi-level CSM (defined as ≥3 levels) remains controversial. Canal decompression can be achieved by posterior strategies; however, the degree of decompression may be insufficient because ventral compression is not resolved [1]. Long-term postoperative axial neck pain may result from surgical invasion of the cervical muscle–ligament complex, and the incidence of C5 nerve root palsy is higher after posterior than anterior surgery due to spinal cord drift [2].

Anterior strategies are effective for neural decompression, especially in cases with preoperative kyphosis, showing better clinical outcomes with less surgical trauma compared to posterior approaches. Traditionally, anterior plating has been used to increase fusion rates and reduce subsidence and postoperative kyphosis. However, complications include screw loosening, screw pullout, dysphagia, and esophageal rupture, especially in multi-level disease [3]. Anterior cervical discectomy and fusion (ACDF) is increasingly performed using stand-alone cages, which overcome the limitations associated with anterior plates and screws [4].

Previous studies showed that ACDF for 3-level CSM using stand-alone cages is technically simpler and involves a shorter operative time, less blood loss, and a lower risk of postoperative dysphagia compared to plate fixation, with satisfactory clinical and radiographic outcomes [5, 6]. To our knowledge, no previous studies have compared the outcomes of ACDF for 4-level CSM using stand-alone cages to laminectomy plus lateral mass fixation and fusion.

## Methods

### Patient population and indications

Patients from a single institution who underwent cervical decompressive surgery for CSM between January 2012 and January 2014 were eligible for this study. Inclusion criteria were: 1) signs and symptoms of CSM not responsive to conservative therapy; 2) age between 18 and 65 years; 3) disc herniation confirmed by computed tomography (CT) or magnetic resonance imaging with spinal cord compression at 4 contiguous disc levels between C3 and C7 and compression limited to the disc level; and 4) ≥ 24 months of follow-up data. Exclusion criteria were: 1) continuous or combined ossification of the posterior longitudinal ligament; 2) developmental stenosis; 3) history of cervical spine trauma and previous cervical spine surgery; or 4) osteoporosis.

Ethical approval was obtained from the institutional review board (No.S043), and written informed consent was obtained from all study participants.

### Surgical procedure

**Fig. 1:**
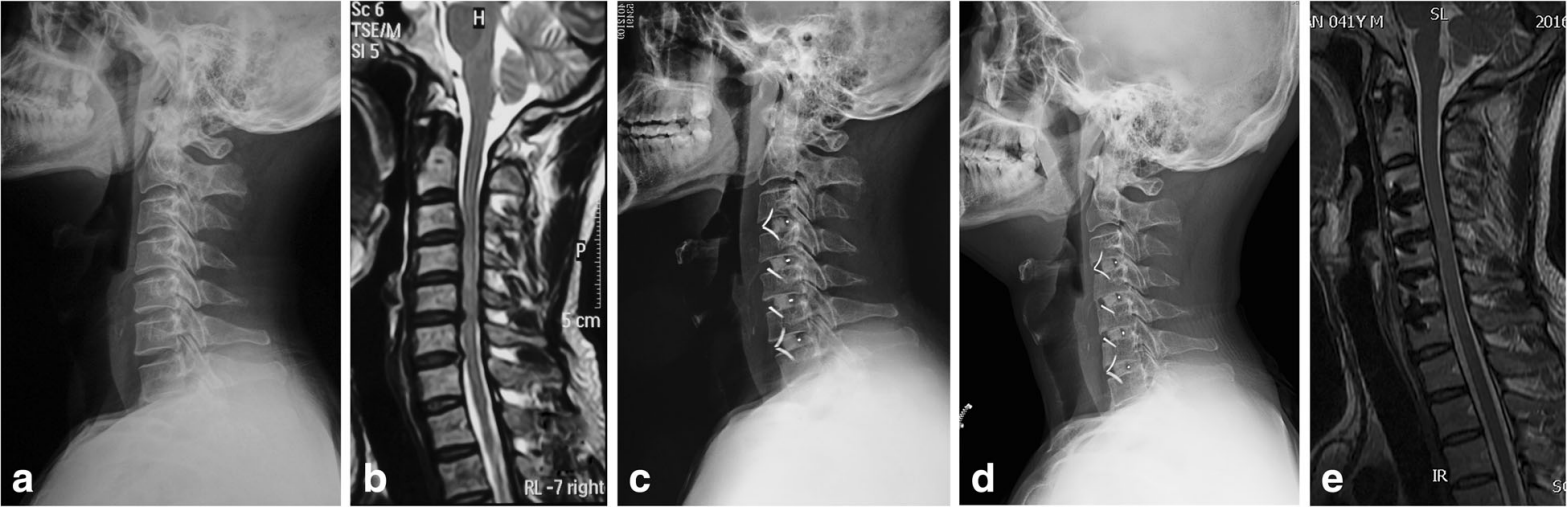
An illustrative case: A 40-year-old male patient who presented with cervical spondylotic myelopathy was treated by 4-level ACDF using a stand-alone cage. **a** Preoperative lateral radiograph of the cervical spine. **b** Disc herniation at C3/4, C4/5, C5/6, and C6/7 confirmed by MRI. **c** Postoperative radiograph showed the cages were well-positioned and cervical spinal alignment was satisfactory. **d** Postoperative lateral radiograph of the cervical spine at the last follow-up showing maintenance of cervical alignment and restoration of disc height. **e** Postoperative MRI at the last follow-up showed thorough decompression of the spinal cord

All surgical procedures were conducted by the same senior surgeon (Kuang). In Group A, ADCF was performed using the standard Smith-Robinson approach [7]. The cartilaginous disc endplate was removed, and care was taken to avoid excessive damage to the bony endplate. Posterior osteophytes were removed by curettes and Kerrison rongeurs. After complete decompression of the spinal cord and nerve roots, radiographic-guided trials facilitated selection of correctly sized cages, as previously described [5]. The distance between the two Luschka’s joints determined the cage width. Cages that fit tightly in the disc space without over-distraction of the disc space or facet joints were considered the correct height. Synthesized hydroxyapatite-collagen artificial bone (Bonegold^®^, Allgens, Beijing, China) was used to fill in the cage in all patients. Properly sized devices (ROI-C^®^ or ROI-MC^+®^, LDR MEDICAL, Troyes, France) and anchorage systems were inserted in vertebral bodies under fluoroscopic guidance (Fig. 1).

In Group B, a midline incision was made followed by a subperiosteal dissection of the paravertebral muscles to expose the spinous processes, laminae, facet joints, and transverse processes of the affected vertebrae. Lateral mass screws (Axon^®^, Synthes Inc., Raynham, MA, USA) were placed on C3-C5 and pedicle screws (Axon^®^, Synthes Inc., Raynham, MA, USA) were placed on C7, respectively.Contoured rods (Axon^®^, Synthes Inc., Raynham, MA, USA) were attached to the screws and locked. Radiographs were obtained to ensure accurate positioning of the screws and rods. Then, the laminae of the planned resection segments were resected using a rongeur and high-speed bur. Prophylactic C4–5 foraminotomy were carried out in patients with foraminal stenosis.Small wedges of auto-graft from the lamina were placed adjacent to bilateral joints to facilitate fusion.

### Outcome assessment

Operative time and blood loss were obtained from medical records. Postoperative follow-up visits were performed at 1 week, 3 months, 6 months, 12 months, 18 months, and 24 months after surgery, and every year thereafter. Clinical outcomes were assessed using Japanese Orthopedic Association (JOA) and Neck Disability Index (NDI) scores. Perioperative and postoperative complications, including hardware-related complications, pseudarthrosis, cage subsidence, dysphagia, C5 palsy, axial neck pain, infection were recorded. Cases with reoperation or revision surgery were also recorded. Radiological outcomes, including fusion rate, cervical lordosis, fused segment lordosis, and cage subsidence, were assessed. Nonunion was defined as the presence of a radiolucent gap between the graft and the end plate on radiographs or CT scans in at least one operated level at the last follow-up [8]. Cervical lordosis was defined as the angle formed by the inferior end plate of C2 and C7 in the neutral position on a plain lateral film [9]. Fused segment lordosis was defined as the angle formed by the superior end plate of C3 and inferior end plate of C7 in the neutral position on a plain lateral film. Improvement in lordosis was defined as an increase in lordosis angle at 1 week after surgery compared to pre-operation. Loss of lordosis was defined as a decrease in cervical lordosis or disc height at the last follow-up compared to 1 week after surgery. Subsidence was defined as loss of > 3 mm disc height at the last follow-up compared to 1 week after surgery [10].

### Statistical analysis

All statistical analyses were conducted using SPSS version 19.0 software (SPSS Inc., Chicago, IL, USA). Between-group clinical and radiological outcomes were analyzed using an independent-samples t test. Within-group comparisons of pre- and post-operative parameters were evaluated using a one-way analysis of variance (ANOVA). Categorical variables, such as the incidence of dysphagia and the complication rate, were assessed using the Chi-square test. *P* < 0.05 was considered statistically significant.

## Results

Medical records of 58 patients who met all the inclusion criteria and none of the exclusion criteria were reviewed. Of these, 26 patients (12 male, 14 female; mean (±SD) age 55.3 ± 10.1 years) underwent ACDF (Group A) with a stand-alone cage, and 32 patients (16 male, 16 female; mean age 54.4 ± 11.7 years) underwent posterior laminectomy and fixation (Group B). There were no significant differences in demographic variables, including patients’ preoperative cervical curvature or JOA and NDI scores, between Groups A and B. Mean follow-up times were 25.1 ± 7.3 months and 25.8 ± 8.9 months in Groups A and B, respectively.

### Clinical outcomes

There was significantly less blood loss in Group A (163.4 ± 72.1 ml) compared to Group B (241.0 ± 112.3 ml) (*P* < 0.05). The operative time was similar in both groups (Group A, 138.7 ± 40.2 min; Group B, 153.3 ± 35.1 min) (Table 1). Both groups demonstrated improvements in JOA and NDI scores after surgery with no significant differences between groups. There were no significant differences between groups at each post-operative follow up time point (Table 2).

**Table 1:** Baseline characteristics of the study population

	Age (years)	Gender (Male/Female)	Follow-up time (months)	Operation time (minutes)	Blood loss (mL)
Group A	55.3 ± 10.1	12/14	25.1 ± 7.3	138.7 ± 40.2	163.4 ± 72.1
Group B	54.4 ± 11.7	16/16	25.8 ± 8.9	153.3 ± 35.1	241.0 ± 112.3
*P*-value	0.78	0.07	0.73	0.15	0.00

Group A underwent four-level ACDF using a stand-alone anchored PEEK cage and Group B underwent four-level laminectomy and fusion

**Table 2:** Clinical outcomes of both groups at different time point

	JOA scores			NDI		
	Group A	Group B	*P*-value	Group A	Group B	*P*-value
Preoperative	10.1 ± 1.1	10.0 ± 1.3	0.80	26.6 ± 3.8	26.8 ± 4.8	0.87
Postoperative 3 month	13.7 ± 1.5 *	13.6 ± 1.5 *	0.80	15.5 ± 1.8 *	15.7 ± 1.6 *	0.68
Postoperative 6 month	13.4 ± 1.3 *	13.3 ± 1.1 *	0.92	14.7 ± 1.7 *	15.0 ± 1.2 *	0.40
Postoperative 12 month	13.2 ± 1.3 *	13.1 ± 1.1 *	0.73	14.3 ± 1.6 *	14.9 ± 1.2 *	0.96
Postoperative 18 month	13.0 ± 1.2 *	13.0 ± 1.0 *	0.92	14.1 ± 1.4 *	14.7 ± 1.2 *	0.10
Postoperative 24 month	12.9 ± 1.2 *	12.8 ± 0.9 *	0.80	14.2 ± 1.4 *	14.4 ± 1.2 *	0.61

Group A underwent four-level ACDF using a stand-alone anchored PEEK cage and Group B underwent four-level laminectomy and fusion

*P-value of the time point versus pre-operation was less than 0.05.

### Radiological outcomes

The fusion rate in Group A and Group B was 86.5 and 100%, respectively. The preoperative cervical lordosis (C2–7) were not significantly different in both groups. The cervical lordosis in both groups improved at postoperative 1 week, 3 months, 6 months, 12 months, but decreased at postoperative 18 months and 24 months (Table 3). Both improvements and loss of lordosis in C2–7 and C3–7 were significantly more pronounced in Group A at the final follow-up (*P* < 0.05). Within each group, there was no significant difference in lordosis improvement between C2–7 and C3–7; however, the loss of lordosis was significantly more pronounced in C2–7 than C3–7 (Table 4).

**Table 3:** Cervical lordosis in both groups at different time point

Cervical Lordosis (C2–7)	Group A	Group B	*P*-value
Preoperative	8.7 ± 3.1	8.7 ± 4.5	0.98
Postoperative 1 week	20.0 ± 4.5*	14.5 ± 6.0*	0.00
Postoperative 3 month	12.8 ± 4.1*	12.5 ± 4.7*	0.78
Postoperative 6 month	10.2 ± 4.3*	10.3 ± 4.9*	0.90
Postoperative 12 month	9.1 ± 4.7*	8.8 ± 5.0*	0.78
Postoperative 18 month	8.5 ± 4.5*	7.0 ± 4.9*	0.59
Postoperative 24 month	8.3 ± 4.6*	7.8 ± 4.9*	0.30

Group A underwent four-level ACDF using a stand-alone anchored PEEK cage and Group B underwent four-level laminectomy and fusion

*P-value of the time point versus pre operation was less than 0.05.

**Table 4:** Improvement and Loss of lordosis in both groups

		C2–7	C3–7	*P*-value within a group
Improvement of lordosis	Group A	11.3 ± 5.9	9.7 ± 5.3	0.32
	Group B	5.8 ± 4.6	5.5 ± 4.5	0.83
	*P*-value between groups	0.00	0.00	
Loss of lordosis	Group A	11.7 ± 2.2	6.7 ± 3.2	0.00
	Group B	7.5 ± 3.8	3.7 ± 3.4	0.00
	*P*-value between groups	0.00	0.00	

Group A underwent four-level ACDF using a stand-alone anchored PEEK cage and Group B underwent four-level laminectomy and fusion

### Complications

The overall complication rate was significantly higher in Group A (15/26) than in Group B (6/32) (*P* < 0.05). There were no instances of perioperative cerebral fluid leakage, hematoma, cage migration, or hardware-related complications in either group. Six patients (14 levels) did not achieve fusion, and 6 patients (16 levels) had cage subsidence at the last follow-up. Patients with pseudarthrosis or cage subsidence were asymptomatic. Three patients in Group A complained of dysphagia post-surgery, but all recovered spontaneously within 3 months. Two patients in Group B complained of pain and paresis of the unilateral deltoid, which was considered to be C5 palsy, within 1 week postoperatively. Patients were treated conservatively using oral neurotrophic drugs, hyperbaric oxygen therapy, and exercise; all patients’ symptoms had resolved by postoperative 3 months. One case of superficial infection was observed in Group B. Three patients in Group B suffered axial neck pain. Symptoms were present at the 6-month follow-up. Two patients had recovered by the final follow-up, while one patient suffered persistent pain. No patients in either group required reoperation **(**Table 5).

**Table 5:** Complications

Subgroup	Group A	Group B
Pseudarthrosis	6	–
Revision surgery	0	0
Hardware-related complications	0	0
Dysphagia	3	0
Infection	0	1
Cage subsidence	6	none
C5 palsy	0	2
Axial neck pain	0	3
Cerebral fluid leakage	0	0
Hematoma	0	0
Total	15	6

Group A underwent four-level ACDF using a stand-alone anchored PEEK cage and Group B underwent four-level laminectomy and fusion

## Discussion

To our knowledge, this is the first study to compare the outcomes of ACDF using a stand-alone cage (Group A) with laminectomy plus fixation and fusion (Group B) for multilevel CSM. ACDF using a stand-alone cage was associated with significantly less blood loss compared to laminectomy plus lateral mass fixation. Operative time was similar for both procedures, and all patients demonstrated improvements in JOA and NDI scores after surgery with no significant difference between procedures. Cervical lordosis and fused segment lordosis were both significantly improved at postoperative 1 week in all patients. However, the loss of cervical lordosis and fused segment lordosis at the final follow-up of 24 months was significantly more pronounced and the complication rate was significantly higher in patients who underwent ACDF using a stand-alone cage.

As a well-established procedure, laminectomy has gained widespread acceptance in the treatment of multilevel cervical myelopathy. Following laminectomy alone, indirect decompression of the anterior aspect of the spinal cord is achieved by spinal cord shift. With posterior fixation, complications associated with multi-level cervical laminectomy, including post-operative kyphosis, segmental instability, and subsequent neurologic deterioration, are reduced [11, 12]. However, adverse outcomes, such as axial pain, C5 palsy, restricted neck motion, perineural adhesions, and late neurologic deterioration, continue to occur.

ACDF is also effective for neural decompression in CSM because it corrects kyphotic alignment and preserves the stability of the cervical spine [13]. Traditionally, anterior plating was used as it was thought to increase fusion rates, reduce cage subsidence, and prevent postoperative kyphosis. However, plate-related complications include screw loosening, screw pullout, dysphagia, and esophageal rupture, especially in long fusions. Ning et al. [14] estimated the rate of anterior cervical plate-related complications at 10.7%. Therefore, ACDF using anterior plate fixation is limited to pathology involving 1 or 2 levels. When spinal cord compression involves more than 3 levels, posterior decompression surgery is advocated. Currently, stand-alone cages are widely used to simplify the surgical procedure and avoid complications related to anterior instrumentation. In accordance with the current study, several reports indicate satisfactory clinical outcomes with ACDF using stand-alone cages for the treatment of multi-level cervical degenerative spondylopathy [5, 6].

Primary concerns in multi-level ACDF using stand-alone cages include nonunion and cage subsidence. Although some clinical studies show relatively high rates of union in multi-level stand-alone ACDF (72–100%) (Table 6) [5, 6, 8, 15], evidence suggests that the risk of pseudarthrosis increases with the number of graft–host interfaces. In the current study, 6 of 26 patients (14 levels) in Group A experienced nonunion; all were asymptomatic and none required revision surgery. This is in accordance with a previous report by Pereira [12] et al. in which three patients experienced late kyphosis due to subsidence and pseudarthrosis; all were asymptomatic, with the exception of one who required revision surgery. The bone substitute in Group A of our study was hydroxyapatite, may be attributed to the relative lower fusion rate to auto-graft in Group B.

**Table 6:** Summary of studies reporting multi-level stand-alone ACDF

Author	Study design	Operated levels	Number of cases	Follow-up time(months)	Device	Fusion rate	Subsidence rate per level (%)
Chen [5]	retrospective	3	28	24	ROI-C or ROI-MC+(LDR MEDICAL, Troyes, France)	85.7%	16.7%
Liu [6]	retrospective	3–4	28	24	ROI-C (LDR MEDICAL, Troyes, France)	100%	–
Pereira [12]	prospective	3–4	30	62	Solis (Stryker, Kalamazoo, MI, USA)	–	16.7%
Liu [16]	retrospective	3	25	24	Solis (Stryker, Cestas, France)	72%	4.0%
Zhou [17]	retrospective	3	15	20	ROI-MC+ (LDR MEDICAL, Troyes, France)	93.3%	8.9%
Our study	retrospective	4	26	24	ROI-C or ROI-MC+(LDR MEDICAL, Troyes, France)	86.5%	15.4%

Cage subsidence is common in multi-level ACDF without anterior plating as the plate curve helps prevent cage subsidence during fusion [16]. Subsidence rates in studies of multi-level ACDF using stand-alone cages range from 8.9 to 16.7% per level [8, 12, 15]. In the current study, the subsidence rate was 6/26 per patient and 13/104 per level in Group A. After reviewing 18 studies of ACDF, including 1468 cases of subsidence, Zajonz et al. suggested that clinical outcomes are unaffected by subsidence [17]. Cage subsidence or loss of disc height may be attributed to loss of cervical lordosis after ACDF. In the current study, patients in group A had a significant improvement in fused segment lordosis at each time point of follow-up compared to pre-operative status. These results are consistent with earlier reports [5, 6]. Postoperative improvements in cervical lordosis and fused segment lordosis were more obvious immediately after surgery in patients in Group A compared to Group B. This suggests that ACDF is more efficacious for restoring cervical disc space and cervical curvature compared to laminectomy and fusion. However, the loss of cervical lordosis at the final follow-up was more pronounced in Group A (11.7 ± 2.2°). This may be due to the use of stand-alone anchored cages. Chen reported that post-operative cage subsidence, loss of disc height, cervical lordosis, and the fused segment angle were relatively higher in multi-level ACDF using stand-alone anchored cages than ACDF using cages and plates [5]. Lee et al. [18] showed that postoperative lordotic changes were 5.85° after ACDF with anterior plating. They proposed that clinical outcomes were unaffected by the loss of cervical lordosis after ACDF. In relation to long-term outcomes, a biomechanical study by Patwardhan et al. showed that cervical spinal sagittal malalignment may play a role in exacerbating adjacent segment degeneration after multilevel fusion as the mechanical burden on the adjacent segment becomes greater due to an imbalanced cervical spine [19]. Therefore, the long-term consequences of cage subsidence and the loss of cervical lordosis after ACDF using a stand-alone cage should be alerted. Interestingly, although improvements in lordosis of the cervical spine (C2–7) and fused segment (C3–7) were similar in both groups, the loss of lordosis was significantly more prominent in the cervical spine (C2–7) than in the fused segment (C3–7). This may be attributed to an acceleration of adjacent segment degeneration in the non-fused C2–3 segment. In that case, measurement of lordosis may not truly reflect the influence of subsidence. As such, the effect of subsidence caused by multi-level ACDF on cervical lordosis may be overestimated.

A previous study shows that C5 nerve root palsy is more likely to happen in patients who undergo laminectomy and fusion compared to ACDF due to excessive spinal cord drift after laminectomy and fusion with preexisting intervertebral foraminal stenosis [20]. A meta-analysis by Shou et al. [2] revealed that the highest prevalence (11.0%) of C5 palsy was found in patients who underwent laminectomy and fusion, while those who received ACDF had the lowest prevalence (3.3%). In the current study, 6.25% patients (2/32) experienced C5 palsy in Group B. Based on the hypothesis of nerve traction and foraminal stenosis as the mechanism of post-laminoplasty C5 palsy, the use of prophylactic C4–5 foraminotomy to decompress the C5 nerve root has been proposed to eliminating this complication. Evidence suggests that prophylactic bilateral C4/C5 foraminotomy significantly decreases the incidence of postoperative C5 palsy (1.4% in the in the foraminotomy group vs. 6.4% in the non-foraminotomy group) [21]; therefore, all patients with foraminal stenosis who underwent laminectomy and fusion in the current study received prophylactic bilateral C4/C5 foraminotomy in our study. However, C5 palsy may still occur in patients with prolonged duration of symptoms and presence of high intensity cord signal changes at C4–5 [22]. In our study, we had two cases (2/32) of post-laminoplasty C5 palsy. Both of them had symptoms for over 1 year.

Axial neck pain is defined as pain from the nuchal to the periscapular or shoulder region after cervical surgery and may result in decreased range of motion. The reported incidence of axial neck pain after posterior cervical spine surgery ranges from 5.2 to 61.5% [23]. Wada et al. [24] found that patients who had undergone posterior procedures complained more frequently of postoperative posterior neck pain than patients who had undergone anterior fusion. Evidence suggests that axial neck pain that occurs within a few months after surgery is due to surgical trauma to muscles, whereas chronic axial neck pain is caused by an imbalance of flexor and extensor muscle strengths [23, 25]. In the current study, 3/32 patients in Group B developed axial neck pain at postoperative 6 months, two of them had recovered by the final follow-up. Only one patient experienced persistent pain. No patients in Group A developed axial neck pain. This may be because anterior approaches avoid posterior muscle invasion and posterior muscle atrophy, which play a pivotal role in the pathogenesis of axial neck pain.

There were limitations in our study. First, it was a non-randomized controlled trial that included a small sample size. The small sample size may reduce the power to detect a difference between the two groups. In addition, the strict inclusion and exclusion criteria of the retrospective study, some cases of cervical canal stenosis or OPLL case had to be treated with posterior approach were excluded, may lead to a potential selection bias. Second, the influence of the graft material on the fusion rate after ACDF using a stand-alone cage was not analyzed. The difference between auto-graft and hydroxyapatite-collagen artificial bone graft in a 4 level ACDF using a stand-alone cage is known. Whether using artificial bone graft in a 4 level fusion without a plate can achieve similar results to traditional ACDF with a plate an auto-graft/allograft is also unknown. Finally, long-term adverse effects require further consideration, such as adjacent segment degeneration resulting from cage subsidence, nonunion, and loss of correction after multi-level ACDF using a stand-alone cage.

## Conclusion

Four-level ACDF using a stand-alone cage appears to be effective compared to laminectomy and fusion when considering clinical outcomes. However, ACDF with a stand-alone cage associated with more non-unions. And loss of correction of cervical lordosis after surgery was more apparent compared to laminectomy and fusion.

## Abbreviations

ACDF: Anterior cervical discectomy and fusion
CSM: Cervical spondylotic myelopathy
JOA: Japanese Orthopedic Association
LF: Laminectomy with fusion
NDI: Neck Disability Index
